# Interlayer Chemical Modulation of Phase Transitions in Two-Dimensional Metal Chalcogenides

**DOI:** 10.3390/molecules28030959

**Published:** 2023-01-18

**Authors:** Zhi Zhang, Yi Wang, Zelin Zhao, Weijing Song, Xiaoli Zhou, Zejun Li

**Affiliations:** 1 School of Physics, Frontiers Science Center for Mobile Information Communication and Security, Southeast University, Nanjing 211189, China; 2 Purple Mountain Laboratories, Nanjing 211111, China; 3 School of Environmental and Biological Engineering, Nanjing University of Science and Technology, Nanjing 210094, China

**Keywords:** interlayer chemical modulation, 2D metal chalcogenides, phase transitions

## Abstract

Two-dimensional metal chalcogenides (2D-MCs) with complex interactions are usually rich in phase transition behavior, such as superconductivity, charge density wave (CDW), and magnetic transitions, which hold great promise for the exploration of exciting physical properties and functional applications. Interlayer chemical modulation, as a renewed surface modification method, presents congenital advantages to regulate the phase transitions of 2D-MCs due to its confined space, strong guest–host interactions, and local and reversible modulation without destructing the host lattice, whereby new phenomena and functionalities can be produced. Herein, recent achievements in the interlayer chemical modulation of 2D-MCs are reviewed from the aspects of superconducting transition, CDW transition, semiconductor-to-metal transition, magnetic phase transition, and lattice transition. We systematically discuss the roles of charge transfer, spin coupling, and lattice strain on the modulation of phase transitions in the guest–host architectures of 2D-MCs established by electrochemical intercalation, solution-processed intercalation, and solid-state intercalation. New physical phenomena, new insight into the mechanism of phase transitions, and derived functional applications are presented. Finally, a prospectus of the challenges and opportunities of interlayer chemical modulation for future research is pointed out.

## 1. Introduction

Along with the isolation of graphene, 2D materials has gain much attention due to their extraordinary properties and a wide array of applications [1,2]. Atomically thin 2D materials present altered electronic structure and crystallographic symmetry distinct from their bulk counterparts, which results in unprecedented physical properties and revolutionary technological advances [3,4,5,6]. More importantly, 2D materials possess a high specific surface area with the ultimate exposure of atoms, which can provide a large number of sites for surface modification to regulate intrinsic properties. Surface modification with chemical species (atom, ions, molecules) can induce charge doping, spin coupling, and lattice strain in 2D materials, tailoring their electronic structures and magnetic interactions, whereby new properties and functionalities are created [7,8,9,10,11,12]. The introduction of surface modification methods has resulted in the research on 2D materials flourishing further, which has caused vigourous development in the past decade.

Recently, a new type of surface modification methodology, namely interlayer chemical modulation, has led to renewed interest in the modification and functionalities of 2D layered materials [13,14]. Due to the anisotropic bonding with weak van der Waals (vdW) interactions between layers, 2D layered materials are suitable host materials for various intercalated guest species, including ions and small molecules. There are several key features for interlayer chemical modulation compared with the conventional surface modification approaches. First, interlayer chemical modulation can form highly stable guest–host structures. The guest species are protected in the interlayers of 2D materials, which eliminate the deterioration of guests caused by reactions in the ambient atmosphere. In addition, the guest species insert themselves into the vdW gap without breaking the in-plane covalent bonds, which preserve the pristine host lattice of 2D materials. Second, interlayer chemical modulation enables the regulation of layer spacing by selecting intercalants with different sizes, which present the electronic characteristics of monolayers or few layers in the bulk without exfoliating the layered materials. Third, taking advantage of the vdW gap as the channel for the motion of guest species, local and reversible modulation on the 2D intra-layer framework can be achieved under external fields. In particular, the interlayer modulation of 2D materials provides a confined space for guest–host interactions, greatly expanding the tunability of their orbital hybridisation, spin coupling, and lattice strain, new and unusual properties that can be induced beyond the limits of conventional surface modification methods.

Amongst various 2D layered materials, 2D metal chalcogenides (2D-MCs), a large family with diverse compositions and structures, harbour a wealth of phase-transition behaviour, including superconductivity, charge density wave (CDW), metal–insulator transitions, and magnetic transitions, which have great prospects in future advanced electronic devices [15,16,17,18]. In general, these phase transitions are the result of strong electronic correlations and electron–phonon coupling in the many-body states of 2D-MCs [19,20] which make interlayer chemical modulation an ideal means to regulate their phase transitions. First, the strongly correlated states in 2D metal chalcogenides are vulnerable to the defects and disorder that would mask the intrinsic many-body behaviour [21]. The maintenance of host lattices and the protection of guest species by interlayer chemical modulation can minimise the introduction of defects and disorder in the processes of modification and functionalities; thus the phase transitions, especially, some susceptive many-body states, can be manifested. Second, the correlated 2D-MCs exhibit exponential sensitivity to the local electronic environment, whereby local modulation can have a profound impact on their phase transitions [22]. Interlayer chemical modulation can afford the opportunities of local and reversible modulation on the basic planes of 2D-MCs where exotic-phase coexistence and evolution can be created. Finally, phase transitions in 2D-MCs are strongly controlled by the charge density, spin-exchange interactions, and lattice configuration [23]. The unique guest–host interactions of confined interlayer modulation can trigger new exciting phase-transition behaviour. Therefore, interlayer chemical modulation is a new and effective way to regulate phase transitions and to explore their complex mechanism and functional applications in 2D-MCs.

In this review, we focus on recent advances in the interlayer chemical modulation of the phase transitions of 2D-MCs, including electronic phase transitions, magnetic phase transitions, and lattice transitions. We mainly review the electrochemical intercalation, solution-processed intercalation, and solid-state intercalation to introduce the guest species into the layered 2D-MCs. We try to clarify the roles of charge transfer, spin coupling, and lattice strain from interlayer guest–host interactions in tailoring the electronic structure, magnetic exchange interactions, and lattice distortion of 2D-MCs. These results demonstrate new phenomena (e.g., coexistence of superconductivity and ferromagnetism) and new functionalities (e.g., neuromorphic computing), as well as the deep understanding of these phase transitions in 2D-MCs. A brief overview of challenges in and the outlook of the interlayer chemical modulation of the phase transitions in 2D-MCs is given at the end.

## 2. Interlayer Chemical Modulation of Electronic Phase Transitions

### 2.1. Superconducting Transition

Superconductivity is a macroscopic quantum phenomenon, which features elusive electron pairing with a zero-resistance state, presenting a unique platform to study strongly correlated physics and to construct advanced electronic devices. The modulation of superconducting states is beneficial for understanding the mechanism of superconducting transitions and for exploring new exciting physical properties. Numerous layered 2D-MCs have demonstrated superconductivity behaviour, providing an ideal model system to modulate the superconducting properties [24]. As is known, superconductivity is sensitive to the charge carrier density and magnetic interactions. It can be expected that interlayer chemical modulations enable stronger host–guest interaction in the confined vdW space, giving rise to enhanced electronic coupling and producing new phenomena.

**Figure 1:**
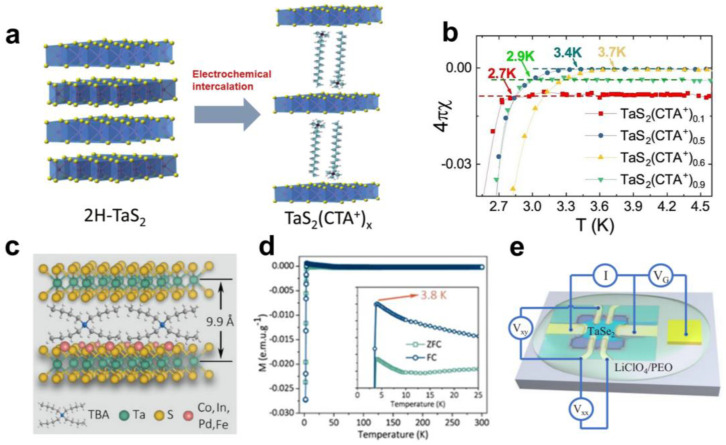
(**a**) Schematic of the crystal structure of 2H-TaS_2_ before and after electrochemical intercalation. Reproduced with permission from Ref. [25]. Copyright 2018, IOP Publishing Ltd. (Bristol, UK). (**b**) Temperature dependence of the magnetic susceptibility of TaS_2_(CTA^+^)_x_. Reproduced with permission from Ref. [25]. Copyright 2018, IOP Publishing Ltd. (**c**) Schematic diagram of TaS_2_ molecular superlattice with metal–organic co-intercalation layer. Reproduced with permission from Ref. [26]. Copyright 2020, Wiley−VCH. (**d**) M−T curves of TaS_2_ molecular superlattices. Reproduced with permission from Ref. [26]. Copyright 2020, Wiley−VCH (Weinheim, German). (**e**) Schematic diagram of the gated intercalation device for 2H-TaSe_2_. Reproduced with permission from Ref. [29]. Copyright 2019, American Physical Society (College Park, MD, USA).

Recently, Wang et al. found the tunable superconducting transition temperature for cetyltrimethylammonium (CTA^+^)-intercalated 2H-TaS_2_ [25]. A series of TaS_2_-(CTA^+^)_x_ (x denotes the intercalated amount of CTA^+^) compounds were obtained by controlling the charge time using cetyltrimethylammonium bromide (CTAB) as the electrochemical intercalation agent (Figure 1a). Through resistivity and Hall measurements, they discovered that the superconducting transition temperature (T_c_) of TaS_2_-(CTA^+^)_x_ first increased and then decreased with the increase of x, displaying a dome-like behaviour. As a result, the T_c_ enhanced from 0.8 K in pristine 2H-TaS_2_ to a maximum of 3.7 K in TaS_2_-(CTA^+^)_x_ (x value is 0.6) (Figure 1b). Furthermore, as the intercalation progressed, the charge carrier concentration of 2H-TaS_2_ near T_c_ increased, indicating that the improvement of T_c_ was closely associated with the increase in the carrier concentration in the superconducting layers. Thus, the charge transfer from CTA^+^ to the TaS_2_ layers may be primarily responsible for the enhanced superconductivity in TaS_2_-(CTA^+^)_x_. Recently, Li et al. developed a method called interlayer-space-confined chemical design (ICCD) that utilises the co-intercalation of organic molecules and Co^2+^ ions to obtain a 2H-TaS_2_ inorganic–organic molecular superlattice with quasi-monolayer properties (Figure 1c) [26]. Magnetic measurements show that the TaS_2_ hybrid superlattices exhibit enhanced superconductivity with the T_c_ of 3.8 K (Figure 1d), which is higher than that of the previously reported monolayer TaS_2_ [27]. They believe that the intercalation of organic macromolecules leads to electronic decoupling between the layers of TaS_2_, which makes the bulk TaS_2_ molecular superlattice exhibit quasi-monolayer characteristics, and this molecular superlattice structure has higher environmental stability than single-layer TaS_2_. In addition, Wu et al. also used a gated intercalation method to insert lithium ions into a 2H-TaSe_2_ flake (Figure 1e), achieving a significant increase in T_c_ from 0.15 K to nearly 2 K [28,29]. The electron doping and interlayer coupling introduced by lithium ion intercalation enhance the electron–phonon interaction, resulting in the enhancement of superconductivity. This demonstrates that interlayer chemical modulation is a facile and effective approach for controlling the electrical properties of 2D materials. It may open up a new avenue for discovering high-temperature superconductors.

**Figure 2:**
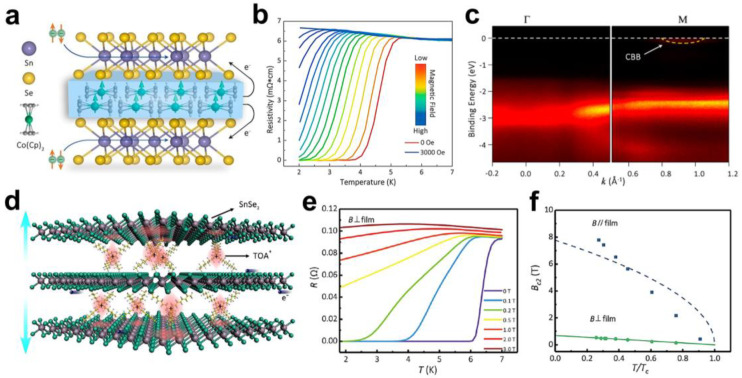
(**a**) Schematic diagram of a 2D organic−inorganic SnSe_2_-Co(Cp)_2_ superlattice structure. Reproduced with permission from Ref. [30]. Copyright 2017, American Chemical Society (Washington, DC, USA). (**b**) Temperature dependence of the resistivity of 2D SnSe_2_-Co(Cp)_2_ superlattices at different magnetic fields. Reproduced with permission from Ref. [30]. Copyright 2017, American Chemical Society. (**c**) The SnSe_2_-Co(Cp)_2_ superlattice energy band structure shown by ARPES. Reproduced with permission from Ref. [30]. Copyright 2017, American Chemical Society. (**d**) Schematic diagram of the crystal structure of (TOA)_x_SnSe_2_. Reproduced with permission from Ref. [31]. Copyright 2021, American Chemical Society. (**e**) Temperature dependence of the resistance in an out-of-plane magnetic field. Reproduced with permission from Ref. [31]. Copyright 2021, American Chemical Society. (**f**) Temperature dependence of in-plane critical magnetic field and out-of-plane critical magnetic field. Reproduced with permission from Ref. [31]. Copyright 2021, American Chemical Society.

More interestingly, interlayer chemical modulation can also induce superconductivity in non-superconducting materials, offering an alternative opportunity to create new types of superconductors. For example, superconductivity was successfully achieved in SnSe_2_ by the intercalation of Co(Cp)_2_ molecules, wherein the pristine SnSe_2_ is a semiconductor [30]. The intercalation of Co(Cp)_2_ molecules into SnSe_2_ was carried out by a solution-processed approach, forming a SnSe_2_-Co(Cp)_2_ organic–inorganic hybrid superlattice (Figure 2a). Through resistivity and magnetic measurements, Li et al. observed that the superconducting T_c_ of the SnSe_2_-Co(Cp)_2_ superlattice was around 5 K, and it exhibited type-II superconducting behaviour (Figure 2b). Angle-resolved photoemission spectroscopy (ARPES) measurements showed that the original Fermi energy level of SnSe_2_ rose across the conduction band, suggesting that the superconductivity of SnSe_2_-Co(Cp)_2_ was due to an increase in the density of electronic states near the Fermi energy by virtue of the electrons transferring from the interlayer Co(Cp)_2_ molecules to the SnSe_2_ layers (Figure 2c). In addition, the isothermal magnetisation curve (M−H curve) was an “S” shape, accompanied by a clear magnetic hysteresis loop, reflecting the ferromagnetic character in the SnSe_2_-Co(Cp)_2_ superlattice. Since the pristine SnSe_2_ is nonmagnetic and Co(Cp)_2_ molecules are paramagnetic, the ferromagnetic behaviour results in the formation of coupled magnetic interactions in SnSe_2_-Co(Cp)_2_ superlattices. Electron spin resonance (ESR) spectra showed that the spatial confinement effect weakened the coordination field of the Co(Cp)_2_ molecules, giving rise to the spin-state transition from a low spin state to a high spin state, thereby resulting in ferromagnetism. This is the first freestanding coexistence of superconductivity and ferromagnetism constructed by non-superconducting and non-ferromagnetic materials of SnSe_2_ and Co(Cp)_2_ molecules, which results in strong interfacial electronic coupling. As a result, they found a unique correlated behaviour of the Kondo effect at low temperature. This coexistence of superconductivity and ferromagnetism in a SnSe_2_-Co(Cp)_2_ superlattice provides an efficient material platform to study the interplay of superconductivity and ferromagnetism, as well as application in superconducting spintronics.

Similarly, the superconductivity of bulk SnSe_2_ could also be achieved by introducing charge into SnSe_2_ via inserting tetraoctylammonium (TOA^+^) cations [31]. The SnSe_2_-(TOA^+^)_x_ superlattice was constructed by the electrochemical intercalation of TOA^+^ into bulk SnSe_2_ (Figure 2d). Resistivity and magnetic measurements showed that the SnSe_2_-(TOA^+^)_x_ superlattice exhibited a superconducting transition at 6.6 K, whilst it showed metallicity above the critical temperature (Figure 2e). In addition, the temperature dependence and the magnetic-field dependence of the resistance are quite different for different magnetic field orientations, suggesting that the SnSe_2_-(TOA^+^)_x_ superlattice behaviour shows anisotropic superconductivity (Figure 2f). Furthermore, below 10 K, as the temperature decreases, the exponential α of the voltage–current–power-law fitting gradually exceeds 1 and increases rapidly, indicating the occurrence of a Berezinskii–Kosterlitz–Thouless (BKT) transition, which evidences the quasi-2D superconducting properties of the SnSe_2_-(TOA^+^)_x_ superlattice. The red shift of the in-plane vibrational mode (E_g_) and the out-of-plane vibrational mode (A_1g_) of SnSe_2_ and the increase in the E_g_/A_1g_ intensity ratio in Raman spectra indicated that the intercalation of TOA^+^ cations into SnSe_2_-induced electron doping thereby led to superconductivity. Moreover, the interlayer spacing of SnSe_2_ was enlarged after intercalation, which greatly weakened the interlayer coupling interactions. However, since the coherence length was larger than the interlayer spacing of the superconducting SnSe_2_ layer, the SnSe_2_-(TOA^+^)_x_ superlattice exhibited a combination of 2D and 3D superconducting properties. More interestingly, the quantum Griffith singularity appeared in the SnSe_2_-(TOA^+^)_x_ superlattice. The random distribution of intercalated TOA^+^ led to the non-uniform charge transfer in the system, which introduced charge disorder into SnSe_2_ layers and triggered the quantum Griffith singularity in the superconducting–metal transition. The organic–inorganic superlattices provides a promising potential platform to investigate the interaction between disordered systems and quantum phase transitions.

**Figure 3:**
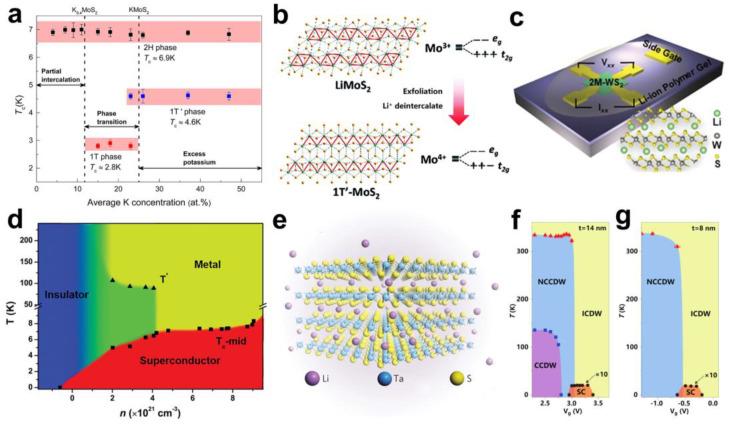
(**a**) K_x_MoS_2_ superconducting structural phase that varies with K content. Reproduced with permission from Ref. [32]. Copyright 2016, American Chemical Society. (**b**) Schematic diagram of the formation of Mo-Mo zig-zag chains in 1T′-MoS_2_. Reproduced with permission from Ref. [34]. Copyright 2017, Royal Society of Chemistry (London, UK). (**c**) Schematic diagram of the 2M-WS_2_-based gated lithium ions intercalation device. Reproduced with permission from Ref. [37]. Copyright 2019, Wiley−VCH. (**d**) Carrier density−temperature phase diagram of 2M-WS_2_ with lithium ion intercalation. The transition temperature T* is an unknown phase transition. Reproduced with permission from Ref. [37]. Copyright 2019, Wiley−VCH. (**e**) Schematic diagram of the lithium ion intercalation structure of 1T-TaS_2_. Reproduced with permission from Reference [40]. Copyright 2015, Springer Nature (Berlin, German). (**f**,**g**) Temperature−gated voltage phase diagrams of 1T-TaS_2_ at 14 nm (**f**) and 8 nm (**g**). Reproduced with permission from Ref. [40]. Copyright 2015, Springer Nature.

As a widely studied layered material, MoS_2_ can also exhibit superconductivity by intercalation with alkali or alkali-earth metals. For instance, Grigorieva et al. discovered that potassium (K) intercalation endowed two metallic 1T polytypes of MoS_2_ with superconductivity [32]. K intercalation was achieved by putting the platelet-shaped 2H-MoS_2_ into a liquid ammonia solution of K metal, and 1T or 1T′ phase KMoS_2_ was obtained depending on the intercalation times. Through energy-dispersive spectroscopy (EDS), X-ray diffraction (XRD), X-ray photoelectron spectroscopy (XPS) characterisation, and magnetisation measurements, they found that, with the increase in K content, MoS_2_ exhibited three kinds of superconducting phases, evolving from 2H-K_0.4_MoS_2_ to 1T-KMoS_2_ and 1T’-KMoS_2_ (Figure 3a). The superconducting T_c_ of these three phases were 6.9 K, 2.8 K, and 4.6 K, respectively, and all three phases showed type II superconductivity. The appearance of superconductivity is induced by the charge doping arising from the electron doping by K atoms. Interestingly, the well-separated superconductivity indicated the coexistence of local order and multi-structural phase. This may provide a basis for the construction of miniature Josephson junctions.

If the above sample is exposed to air, oxygen and water react with the intercalated K, causing the K to de-intercalate, which is similar to the results of previous studies [33]. Surprisingly, they found that, after K_x_MoS_2_ was exposed to air for de-intercalation, the XPS results showed that the structural changes induced by K intercalation still existed, but the superconducting signal disappeared. This seems to indicate that the structural changes brought by the intercalation do not contribute to the generation of MoS_2_ superconductivity. However, the inevitable multiphase-coexistence structure of MoS_2_ in the above experiments makes it impossible to clearly study the contribution of a single structural change to the superconducting phase transition. Therefore, it is of great significance to obtain pure 1T or 1T′-phase MoS_2_. Guo et al. discovered intrinsic superconductivity in pure-phase 1T’-MoS_2_ produced by the structural phase transition of intercalated 2H-MoS_2_ [34]. They fabricated highly crystalline LiMoS_2_ by intercalating lithium uniformly into 2H-MoS_2_ through modified high-temperature solid-state reactions. The charge transfer caused by the lithium between the layers induced a valence change of Mo in 2H-MoS_2_, resulting in a uniform octahedral coordination structure. This highly ordered intercalation structure obtained by lithium intercalation is a prerequisite for the formation of 1T’-MoS_2_. Following liquid-phase exfoliation in distilled water and lithium removal to obtain monolayer MoS_2_, the Mo^3+^ in the original octahedra was oxidised to Mo^4+^, which transformed all of the diamond-like Mo-Mo chains into zig-zag chains and formed a long-range ordered distorted octahedral coordination structure, eventually yielding pure 1T′-MoS_2_ (Figure 3b). The resistivity and magnetic measurements showed that 1T′-MoS_2_ had type II superconducting properties and a superconducting transition at 4.6 K, which were absent in pure 2H phase. These results demonstrated that the twisted octahedral structure of 1T′-MoS_2_ brings about extraordinary electronic interactions, which in turn trigger superconductivity. In addition, Fang et al. also successfully prepared pure bulk 1T-MoS_2_ using the intercalation compound of LiMoS_2_ and found that it also possessed intrinsic superconductivity with a superconducting T_c_ of 4 K [35]. These findings provide a new avenue for exploring the mechanism of the superconducting transition and also showed that the structural phase transition induced by intercalation played a critical role in the study of the intriguing properties of various layered materials.

As discussed above, various structural phases, such as metastable 1T/1T′ phases and the stable 2H phase, have been discovered in transition-metal dichalcogenides (TMDs). The physical and electronic properties of TMDs are substantially governed by their structural phases. 2M-WS_2_ is a new structural phase of TMDs recently reported. Pristine 2M-WS_2_ exhibits a metallic behaviour and is a metastable p-type intrinsic superconductor. The superconducting T_c_ of 2M-WS_2_ was 8.8 K, which was the highest intrinsic superconducting T_c_ in TMDs [36]. Afterwards, Che et al. found a reversible superconducting–insulating transition in electron-doped 2M-WS_2_ [37]. The 2M-WS_2_ flakes were obtained by mechanical exfoliation, and the lithium ions were intercalated into the layers of 2M-WS_2_ by applying gate voltage (V_g_) (Figure 3c). Raman spectroscopy and Hall measurements revealed that the carrier concentration of the sample decreased from 9.05 × 10^21^ cm^−3^ to −6.04 × 10^20^ cm^−3^ as the V_g_ increased. Meantime, the resistance measurement showed that, with the increase in V_g_, the superconducting T_c_ of the sample decreased, the resistance increased rapidly, and, finally, the sample transformed from a superconducting state to a completely insulating state, which can be clearly seen in the phase diagram (Figure 3d). This is because the electron doping arising from lithium ion insertion leads to a significant decrease in the concentration of majority carriers (holes) in 2M-WS_2_, thereby suppressing superconductivity and producing an insulating state. In addition, the decrease in T_c_ caused by a decrease in the majority carrier density is similar to a phenomenon recently found in Cu-based superconductors [38]. In Cu-based superconductors, the change in the superconducting plane buckling angle caused by doping elements would affect T_c_ [38,39]. Therefore, it is speculated that the buckling angle on the 2M-WS_2_ superconducting plane also might be adjusted by lithium ion doping, which affects its superconducting T_c_. It is worth mentioning that the superconducting–insulating transition in 2M-WS_2_ was reversible. By applying gate voltage to de-intercalate lithium ions, the original superconductivity of the sample could be restored. This indicates that gate-controlled intercalation is of great importance for the study of the complex electronic states induced by charge doping.

The formation of superconductivity by charge doping via gate electric field is usually effective for non-superconductors with low charge carrier density. However, in terms of materials with a high carrier concentration, it is difficult to control their phase transitions, especially the superconducting transition. For example, 1T-TaS_2_ is a layered TMD with complex charge ordered states. Due to its high carrier concentration, it is difficult to control its phase transition by conventional gate modulation. To address this challenge, Yu et al. reported multiple phase transitions from Mott insulator to superconductor and then to metal in a high-charge-doped 1T-TaS_2_ flake with the aid of a new intercalation method [40]. The high-charge-doped 1T-TaS_2_ was obtained by an ionic field effect transistor (iFET) based on 1T-TaS_2_ in the case of gate-controlled lithium ion intercalation (Figure 3e). On the one hand, they found that several charge density wave (CDW) phase transitions of undoped 1T-TaS_2_ were modulated by the sample thickness. On the other hand, in the bulk limit (thickness > 10 nm), with the increase in V_g_, the charge doping gradually increased, the Mott state in 1T-TaS_2_ disappeared with the disappearance of the commensurate CDW (CCDW) phase, and a superconducting phase with T_c_ of 2 K appeared at the phase transition intersection of the nearly commensurate CWD (NCCDW)/the incommensurate CDW (ICWD) phase (Figure 3f). In the quasi-2D (thickness < 10 nm) 1T-TaS_2_, the CCDW phase did not exist due to the decrease in dimensionality, but the superconducting–metal transition still existed in the sample as in the bulk (Figure 3g). Whilst in the 2D limit (~3 nm), the charge doping partially suppressed the original insulating state of the 2D sample, which may be due to disorder. In the charge-doped 1T-TaS_2_, the microscopic phase separation at the junction of the NCCDW/ICDW phase transition may provide electron–phonon coupling for the formation of Cooper pairs, which may be the cause of superconductivity. It can be expected that gate-controlled-ion intercalation techniques provide powerful tools for interaction studies in the presence of extreme carrier doping and the design of 2D devices.

In general, the charge doping and interlayer coupling interaction caused by different intercalation elements are different, which will have different effects on the electronic properties of the host materials. However, Zhang et al. recently found almost identical superconducting T_c_ and electronic properties in a series of alkali and alkali-earth metal intercalated black phosphorus (BP) crystals [41]. Their study found that the phosphorene layer could always maintain almost the same electron doping density as the intercalation of Li, K, Rb, Cs, Ca metal atoms. This extraordinary superconducting behaviour indicated a more complex interaction mechanism, which was very different from that of MCs. These findings illustrate the great potential of the intercalation method in the regulation of superconductivity and provide a new method for further studying the underlying mechanism of abnormal superconductivity.

### 2.2. Charge Density Wave Transition

As a quantum-ordered state, CDW phase transition often appears in low-dimensional materials with strong electron correlation interactions and has complex interactions with the superconducting phase, Mott insulating phase, and other ordered phases [19,42,43]. Studying the CDW phase transition and its interactions with other electronic states are advantageous for the in-depth understanding of various complex correlation interactions, which is pivotal for the effective regulation of the electronic properties of low-dimensional materials and the application of low-dimensional electronic devices. At present, charge density waves have been found in a variety of material systems, especially in low-dimensional layered MCs, providing a versatile platform for studying the ordered origin of CDW phases. It is well known that layered compounds are susceptible to molecular or atomic intercalation, which is of paramount importance for the generation and regulation of CDW transitions.

**Figure 4:**
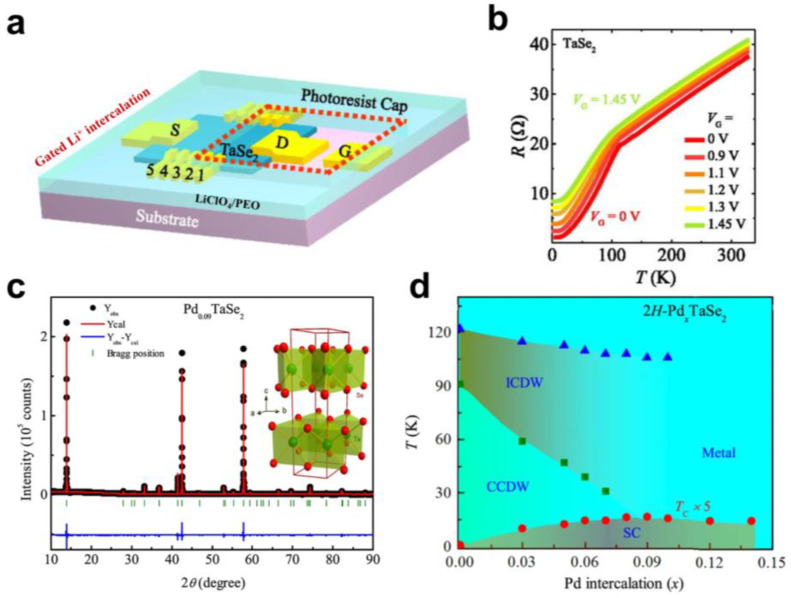
(**a**) Schematic diagram of the gated lithium ion intercalation device for a specific region of 2H-TaSe_2_. Reproduced with permission from Ref. [45]. Copyright 2018, AIP Publishing (Binghamton, NY, USA). (**b**) Temperature dependence of the resistance of TaSe_2_ at different V_g_. Reproduced with permission from Ref. [45]. Copyright 2018, AIP Publishing. (**c**) The PXRD pattern of Pd_0.09_TaSe_2_ shows the 2H phase structure that remains after the intercalation, and the inset is a schematic diagram of the 2H-TaSe_2_ crystal structure. Reproduced with permission from Ref. [49]. Copyright 2016, Springer Nature. (**d**) The temperature–Pb content phase diagram of the intercalated TaSe_2_. Reproduced with permission from Ref. [49]. Copyright 2016, Springer Nature.

The gated intercalation mentioned above is an effective and controllable ion intercalation method, which can be used to realise the modulation of CDW in 2D-MCs and study the microscopic interaction process caused by intercalation. However, for 2D materials, the electric double layer formed by the electrostatic gating effect may interfere with the influence of ion intercalation [44]. Therefore, it is very important to identify the change in material properties caused by electric double layer and ion intercalation. Recently, Wu et al. developed a gating ion intercalation technology combined with lithography technology, studied the microscopic process of lithium ions intercalation 2D 2H-TaSe_2_, and realised the gating intercalation modulation of CDW [45]. By covering a part of the crystal surface with a photoresist, lithium ions were intercalated from the uncovered side, thus distinguishing the electrostatic gating effect from the intercalation effect (Figure 4a). The similar gate voltage dependence of channel resistance in the covered and uncovered regions indicated the dominance of ion intercalation. Furthermore, they found that the hump of the R-T curve gradually disappeared with increasing V_g_, indicating that the CDW transition was suppressed by the lithium ion intercalation (Figure 4b). In addition, the anomaly that the channel resistance increased, then decreased, and then increased again illustrated the complexity of the intercalation process. They suggested that the modulation of CDW and resistance by lithium ion intercalation was not achieved by simple carrier doping but may have been closely related to the position distribution of lithium ions after intercalation.

Generally, the CDW in TMDs is considered to be generated by electron–phonon coupling near the Fermi surface [46]. The superconductivity in most materials is also closely related to electron–phonon coupling. Therefore, there exists a competition between the CDW and the superconductivity in certain materials, which has also been confirmed by many studies. For example, Fang et al. discovered competition between superconductivity and CDW in the Na-intercalated crystal of Na_x_TaS_2_ grown directly through a solid-state reaction [47]. The Na content in the intercalated 2H-TaS_2_ was analysed by the energy-dispersive X-ray microanalysis (EDX) images of a scanning electron microscope (SEM), and both magnetic and resistive properties were measured. It was found that the CDW phase transition gradually disappeared with the increase in Na. Meanwhile, the superconducting phase appeared and increased with the increase in Na, and the maximum superconducting T_c_ reached 4.4 K. This result suggested a competitive relationship between CDW states and superconductivity. Superconductivity appeared mainly due to the increase in the density of states near the Fermi surface when the CDW state was suppressed.

Although the competition between superconductivity and CDW has been found in many materials, the physical mechanism is still poorly understood. The in-depth study of the competition between superconductivity and CDW can help us further understand the physical properties of CDW in 2D materials, guiding the regulation and functionality of materials. Like 2H-TaS_2_, 2H-TaSe_2_ also presents a typical CDW phase transition with intrinsic superconducting properties. The metal/ICDW phase transition, the ICDW/CCDW phase transition, and the CCDW/superconducting transition of 2H-TaSe_2_ can occur with a decrease in temperature [48]. Bhoi et al. found multiband superconductivity and direct competition between CCDW and superconductivity in a Pb-intercalated 2H-TaSe_2_ crystal. 2H-Pd_x_TaSe_2_ crystals were synthesised through a solid-state reaction [49]. The powder X-ray diffraction pattern (PXRD) showed that the c-direction spacing increased due to the intercalation of Pb (Figure 4c). By measuring the resistivity and magnetic properties of Pb_x_TaSe_2_ with different x, they found that, with the increase in x, the transition temperature of the CCDW phase was rapidly suppressed, whilst the transition temperature of the ICDW phase decreased slowly. The superconducting transition temperature exhibits a dome shape with the increase in Pb. When x = 0.9, the superconducting temperature reaches a maximum of 3.3 K, and the CCDW phase collapses (Figure 4d). This indicates that the superconducting phase seems to be directly competitive with the CCDW phase but not directly related to the ICDW. The results of specific heat measurement and calculation analysis indicate that the increase in superconductivity is due to the increase in effective electron–phonon coupling and density of states at the Fermi level due to the embedding of Pb. In addition, the electron-specific heat and the temperature dependence of the critical magnetic field indicate that there are multiple superconducting energy gaps in 2H-Pb_x_TaSe_2_. The different interactions of superconductivity with CCDW and ICDW indicate that the two types of CDW sequences in 2H-TaSe_2_ seem to have different origins. This is similarly demonstrated by recent studies on 2D 2H-TaSe_2_ intercalated by lithium ions. Wu et al. found that Fermi surface nesting and electron–phonon interaction in 2H-TaSe_2_ do not seem to be the main causes of the ICDW order [29]. The controlled lithium ion intercalation of 10 nm thick 2H-TaSe_2_ was performed by an iFET device. The flake 2H-TaSe_2_ was mechanically exfoliated from the 2H-TaSe_2_ crystal prepared by CVT. Through Hall measurement at low temperature, it is found that the lithium-intercalated 2H-TaSe_2_ has a negative Hall coefficient at low gate voltage (V_g_) and exhibits a nonlinear dependence of the magnetic field. At higher V_g_, the Hall coefficient becomes positive and has a linear relationship with the magnetic field. This indicates that the intercalation of lithium ions causes the topological change of the Fermi surface from a multi-pocket to a single-pocket in the ICDW phase. Moreover, the change in the Fermi surface topology and the suppression of the ICDW phase appear in different V_g_, which indicates that the nesting of the Fermi surface is independent of the appearance of ICDW. The Raman spectra of 40 nm-TaSe_2_ before and after intercalation also show that the intercalation has more influence on the characteristic phonon spectrum, which corresponds to the change in the Fermi surface topology rather than the inhibition of ICDW. In addition, through electrical transport measurement and calculation fitting, they found that the electron–phonon scattering in the intercalated sample decreases with the increase in V_g_, which indicates that the combined effect of carrier doping and interlayer coupling caused by lithium ion intercalation leads to the enhancement of electron–phonon interaction. Meanwhile, the obvious increase in the superconducting temperature and the small change in the ICDW phase transition temperature also shows that the electron–phonon interaction in 2H-TaSe_2_ may not be the main cause of ICDW order. Therefore, superconductivity and CCDW in 2H-TaSe_2_ are regulated by the same type of electron–phonon coupling, whilst ICDW order may come from another type of electron–phonon coupling or other unknown mechanisms. In a word, these findings give a deep insight into the physical origin of the interaction between electron phase transitions in 2H-TaSe_2_. It is also demonstrated that interlayer modulation can be an effective means to regulate the delicate balance between various electronic phase transitions.

**Figure 5:**
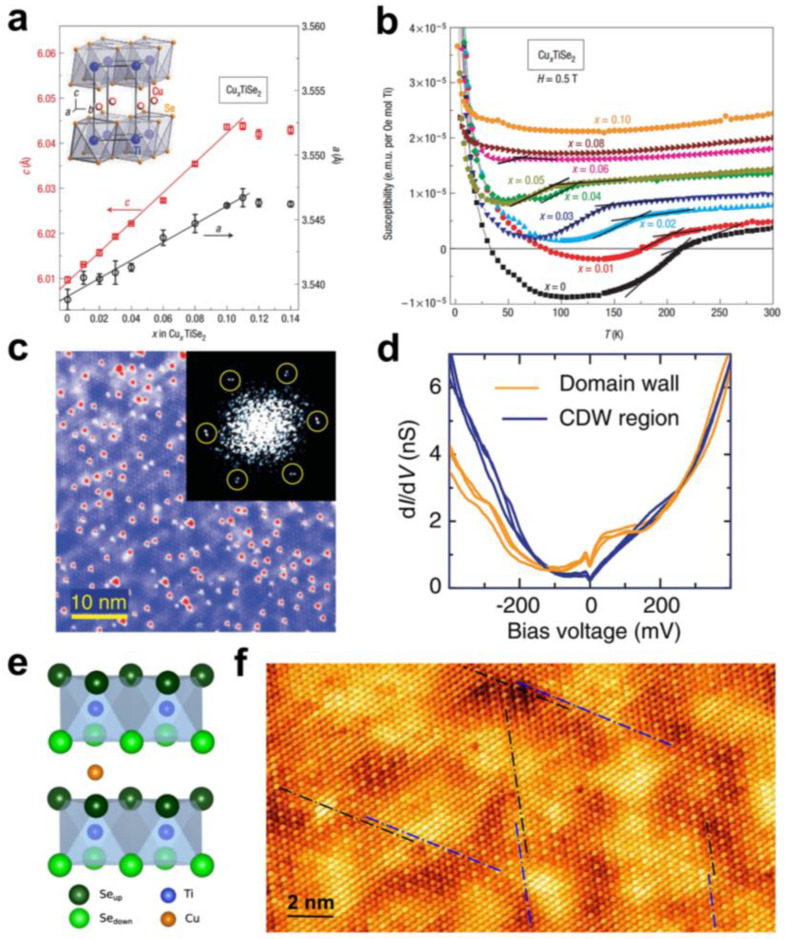
(**a**) The lattice constant of Cu_x_TiSe_2_ varies with the Cu content, and the inset is a schematic diagram of the lattice structure of Cu_x_TiSe_2_. Reproduced with permission from Ref. [51]. Copyright 2006, Springer Nature. (**b**) Temperature dependence of the magnetic susceptibility of Cu_x_TiSe_2_ at 0.5 T. Reproduced with permission from Ref. [51]. Copyright 2006, Springer Nature. (**c**) STM topography on Cu_0.08_TiSe_2_. Reproduced with permission from Ref. [59]. Copyright 2017, American Physical Society. (**d**) STM dI/dV maps for CDW and domain wall regions. Reproduced with permission from Ref. [59]. Copyright 2017, American Physical Society. (**e**) Schematic diagram of the lattice structure of 1T-Cu_x_TiSe_2_. Reproduced with permission from Ref. [60]. Copyright 2017, American Physical Society. (**f**) Short-range ordered CDW domains in Cu_0.07_TiSe_2_. Reproduced with permission from Ref. [60]. Copyright 2017, American Physical Society.

The application of interlayer modulation on single CDW phase with high transition temperature has been demonstrated to produce new physical phenomena [50]. The CDW–superconductivity transition in Cu-intercalated 1T-TiSe_2_ was discovered early by Morosan et al. Cu-intercalated TiSe_2_ was prepared by the solid-phase method [51]. XRD analysis showed that Cu occupied the interlayer of TiSe_2_ (Figure 5a). The CDW state was gradually suppressed with the increase in Cu in Cu_x_TiSe_2_. Interestingly, a superconducting phase appeared in the suppressed CDW state. Through magnetic transport measurements, they found that the CDW phase gradually disappeared with increasing Cu content (Figure 5b). Furthermore, the superconductivity exhibited typical dome behaviour. This is a reflection of the competitive behaviour of CDW and superconductivity. Further studies showed that the suppression of CDW mainly originates from the lattice change due to Cu doping. The disordered intercalation of Cu breaks the superlattice formed by the CDW transition in TiSe_2_, which suppresses the CDW state [52]. The dome behaviour of superconductivity can be understood as follows: Initially, the intercalation of Cu effectively increases the density of the state near the Fermi surface, which enhances superconductivity. However, the strong inelastic scattering caused by excess Cu suppresses the superconductivity [53]. The competition phenomenon of CDW and superconductivity in 1T-TiSe_2_ seems to suggest that the origin of CDW is strongly related to electron–phonon coupling. However, the special band structure of 1T-TiSe_2_ makes it possible to have exciton insulator phase transitions, which may also cause CDW transitions. Therefore, the origin of CDW in 1T-TiSe_2_ is still debated, mainly including exciton condensation [54], electron–phonon interaction [55], and the interaction of excitons and phonons [56,57]. In addition, studies on the gated doping of 1T-TiSe_2_ point out that CDW and superconductivity are not simply in direct competition with each other. Before the appearance of superconductivity, another ICDW phase appeared in the CDW phase, wherein their domain wall might be critical to the formation of Cooper pairs [58]. The ICDW phase accompanying the superconducting phase was also found in the superconducting 1T-Cu_0.08_TiSe_2_ [59]. Yang et al. found that, before reaching the superconducting temperature, an inhomogeneous ICDW phase formed by a CCDW phase shift appeared in the CCDW phase of Cu_0.08_TiSe_2_ by Fourier-transform scanning tunneling spectroscopy (Figure 5c). Further, the study of STM dI/dV maps shows that the formation of ICDW is dominated by electron–phonon coupling caused by Cu intercalation (Figure 5d).

Strikingly, a recent study of TiSe_2_ with Cu intercalation by Novello et al. may contradict the notion that CDW in this material originates from exciton pairing [60]. They prepared 1T-Cu_x_TiSe_2_ single crystals by the iodine vapour transport (IVT) method, and the Cu atoms were distributed in the interlayer of TiSe_2_ (Figure 5e). The charge distribution and Cu atomic distribution in these Cu_x_TiSe_2_ were investigated by scanning tunneling microscopy (STM), and it was found that the charge-ordered distribution was related to the local content of Cu. Symmetry-breaking stripe CDW domains appear at low Cu content (x = 0.01). At high Cu content (x = 0.07), the original long-range ordered CDW superlattice in TiSe_2_ transforms into short-range ordered CDW nanodomains (Figure 5f), which are not reflected in the transport measurements. Thus, this result supports the idea that the main cause of CDW in 1T-TiSe_2_ is not simply exciton pairing. However, the latest ultrafast electron diffraction studies on pristine 1T-TiSe_2_ show that exciton condensation in 1T-TiSe_2_ is directly related to the formation of 2 × 2 2D CDW order within the layers and promotes the transformation of 2D CDW order within the layers to 2 × 2 × 2 3D CDW order between the layers [61].

### 2.3. Semiconductor-to-Metal Transition

**Figure 6:**
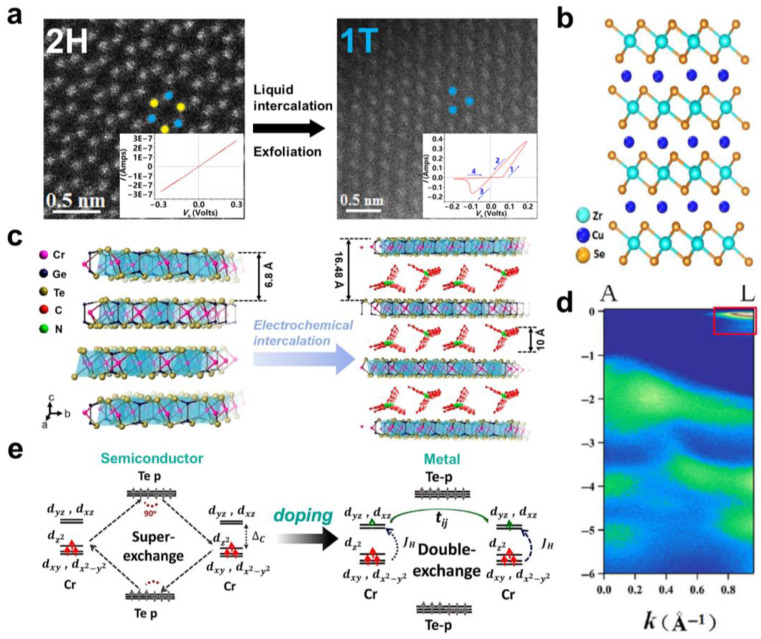
(**a**) The high-angle annular dark-field scanning transmission electron microscopy image of MoS_2_ flake. The blue and yellow dots represent Mo and S atoms, respectively. The inset shows the characteristic curve of I−V at room temperature. Reproduced with permission from Ref. [66]. Copyright 2016, American Chemical Society. (**b**) Schematic diagram of the lattice structure of Cu_0.07_ZrSe_2_. Reproduced with permission from Ref. [67]. Copyright 2018, Springer Nature. (**c**) Schematic diagram of the lattice structure of Cr_2_Ge_2_Te_6_ before and after intercalation. Reproduced with permission from Ref. [68]. Copyright 2019, American Chemical Society. (**d**) The energy band structure of Cu_0.07_ZrSe_2_ shown by ARPES. Reproduced with permission from Ref. [67] Copyright 2018, Springer Nature. (**e**) Schematic diagram of the superexchange interaction in the semiconducting Cr_2_Ge_2_Te_6_ and the double exchange interaction in the metallic (TBA)Cr_2_Ge_2_Te_6_. Reproduced with permission from Ref. [68]. Copyright 2019, American Chemical Society.

In recent years, the study of the semiconductor–metal phase transition of 2D materials has attracted much attention and has demonstrated great prospects in the construction of integrated devices by using their homojunction structures. TMDs usually have an abundance of structural phases, which exhibit different electrical properties. For example, MoS_2_ has a semiconducting 2H phase, a metallic 1T phase, and a 1T’ phase. It has been found that several of these structural phases can be transformed into each other by ion intercalation [62], gated charge doping [63], annealing [64], or plasma induction [65]. Cheng et al. fabricated an ideal odd-symmetric memristor using 1T-MoS_2_ nanosheets [66]. 1T-MoS_2_ nanosheets were prepared by lithium ion intercalation. Through I-V measurements, the Ag/MoS_2_/Ag device exhibited an alternating on–off state and an asymmetric pinched hysteresis loop, indicating that it is an asymmetric switch with memristive behaviour (Figure 6a). Moreover, the 1T-MoS_2_ nanosheets have stable memristive behaviour without perceptible changes after 1000 I-V cycles. In addition, an ideal memristor Ag/MoS_2_/Ag/MoS_2_/Ag with odd symmetry was obtained by combining two asymmetric devices. Its I-V curve is an odd-symmetric “pinched” loop with excellent stability. 1T-MoS_2_ nanosheets exhibit memristive behaviour because the electric field can cause the lattice distortion of nanosheets. When the external electric field is large, Mo and S ions in the lattice shift, resulting in lattice distortion. This lattice distortion enhances electron delocalisation and leads to an increase in conductivity. In contrast, when a reverse electric field is applied, the displaced Mo and S ions are relocated, resulting in a decrease in conductivity.

In addition to the structural phase transition, the change of electronic band caused by interlayer modulation can also lead to semiconductor–metal transition. For example, the original ZrSe_2_ is an indirect band-gap semiconductor. Muhammad et al. realised the semiconductor–metal transition of ZrSe_2_ by Cu intercalation [67]. Cu_0.07_ZrSe_2_ was synthesised by CVT, and Cu was located in the interlayer of ZrSe_2_ (Figure 6b). Through I-V measurements, Cu_0.07_ZrSe_2_ showed a linear I-V curve, whilst the original ZrSe_2_ showed a nonlinear I-V curve, indicating the semiconductor–metal transition of Cu-intercalated ZrSe_2_. The Cu^+^ was evidenced in Cu_0.07_ZrSe_2_ by XPS measurements, indicating that Cu atoms contribute electrons to the ZrSe_2_ layer and form a stable closed-shell Cu^+^. First-principles theory calculations and ARPES measurements suggest that the electronic doping introduced by the Cu intercalation leads to an increase in the Fermi level of ZrSe_2_ crossing the conduction band bottom (Figure 6d), which changes ZrSe_2_ from semiconductor to metal. Further, some layered magnetic semiconductors can also be modified by molecular intercalation. Wang et al. found a transition from ferromagnetic semiconductor to ferromagnetic metal in organic molecular intercalated Cr_2_Ge_2_Te_6_ [68]. The Cr_2_Ge_2_Te_6_ single crystals were synthesised by the flux method, and the organic cation TBA^+^ from the tetrabutylammonium bromide (TBAB) solution was intercalated into the single crystals by an electrochemical process (Figure 6c). Through magnetic and resistivity measurements, they found that the Curie temperature of (TBA) Cr_2_Ge_2_Te_6_ increased from 67 K to 208 K, and the magnetic easy axis was reoriented from the *c*-axis to the ab-plane. More interestingly, the resistance shows a broad peak metal behaviour near 150 K. Theoretical calculations show that the electron doping introduced by the intercalation leads to the semiconductor–metal phase transition. The increase in the Curie temperature is due to the change of the Cr spin magnetic coupling from weak super-exchange mediated ferromagnetism to metal double-exchange ferromagnetism caused by electron doping (Figure 6e). This interlayer chemical modulation provides a new way to control the electronic and magnetic properties of 2D materials.

## 3. Interlayer Chemical Modulation of Magnetic Phase Transitions

**Figure 7:**
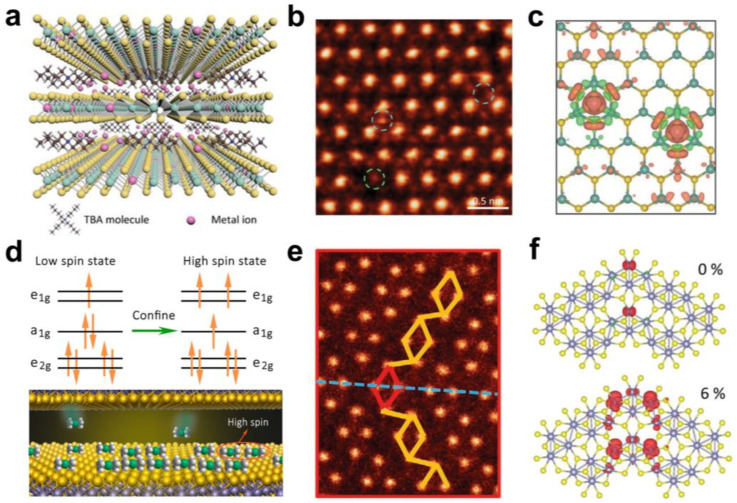
(**a**) Schematic diagram of Co-TaS_2_ molecular superlattice structure. Reproduced with permission from Ref. [26]. Copyright 2020, Wiley−VCH. (**b**) The high-angle annular dark-field scanning transmission electron microscopy image of the surface of a Co-TaS_2_ superlattice flake. Reproduced with permission from Ref. [26]. Copyright 2020, Wiley−VCH. (**c**) Schematic diagram of the spin density of Co atoms in the hollow position of monolayer TaS_2_. Reproduced with permission from Ref. [26]. Copyright 2020, Wiley−VCH. (**d**) Spin state transition of Co(Cp)_2_ molecules between SnSe_2_ layers. Reproduced with permission from Ref. [30]. Copyright 2017, American Chemical Society. (**e**) STEM images show the MTBs in ReS_2_. Reproduced with permission from Ref. [72]. Copyright 2020, Wiley−VCH. (**f**) Schematic diagram of the spin density of S vacancies in MTBs under different strains. Reproduced with permission from Ref. [72]. Copyright 2020, Wiley−VCH.

Magnetic materials are the basis for constructing spintronic devices with high performance, low power consumption, and easy integration. Recently, the discovery of 2D magnetic materials CrI_3_ and Cr_2_Ge_2_Te_6_ has been a great impetus to research in related fields [69,70]. Although many layered materials with expected 2D magnetism have been theoretically predicted, stable intrinsic 2D ferromagnets are still rare, which causes difficulties in studying their fundamental physical properties and spintronic applications [2,71]. Interlayer chemical modulation has recently been experimentally demonstrated to introduce magnetic transition in 2D materials, providing a new way to study 2D magnetism. For example, long-range ferromagnetic coupling was observed in a dilute single-atom-doped TaS_2_ molecular superlattice [26]. The TaS_2_ molecular superlattices were synthesised by the ICCD method. The TaS_2_ crystals were placed into the tetrabutylammonium chloride–dimethylformamide (TBAC–DMF) solution of CoCl_2_ to realise the intercalation of organic molecules and the co-intercalation of Co ions, thus obtaining the alternative Co-TaS_2_ molecular superlattice (Figure 7a). The resistivity and magnetic measurements show that the Co-TaS_2_ superlattice has stable ferromagnetism at both low and room temperatures. The interlayer electron decoupling caused by large intercalated molecules allows the bulk Co-TaS_2_ superlattice to exhibit the electronic characterisation of monolayer TaS_2_. More interestingly, the exfoliated 2D Co-TaS_2_ nanosheets still have ferromagnetic properties. Co atoms form two adsorption configurations by substituting Ta atoms or occupying vacancies on the TaS_2_ surface (Figure 7b). These Co atoms form orbital-specific p-d hybridisation with adjacent Ta and S atoms, resulting in spin polarisation (Figure 7c). The spin polarisation of Co atoms with the indirect RKKY spin-exchange interaction mediated by itinerant carriers in metallic TaS_2_ layers may be responsible for the long-range ferromagnetism in Co-TaS_2_. Similarly, as mentioned above, using a nonferromagnetic organic molecule Co(Cp)_2_ intercalation, a ferromagnetic transition were induced in a 2D SnSe_2_-Co(Cp)_2_ superlattice [30]. The ferromagnetism originates from the high spin state of Co(Cp)_2_ caused by the spatial confinement between the SnSe_2_ layers (Figure 7d).

When electrochemical intercalation is used to exfoliate large-sized nanosheets, lattice strain defects (e.g., dislocations and grain boundaries) arising from large-sized molecular insertions will also affect the properties of 2D materials, resulting in some new interesting phenomena. For example, the ferromagnetic transition has been demonstrated in electrochemically exfoliated monolayer ReS_2_ [72]. The ReS_2_ monolayer was obtained by the electrochemical exfoliation of ReS_2_ crystals using organic macromolecules tetrapropylammonium (TPA) and tetraheptylammonium (THA) as intercalation agents. Compared with the nanosheets less than 10 μm obtained by mechanical exfoliation [73], the ReS_2_ monolayer obtained by electrochemical intercalation and exfoliation has a higher proportion and larger size. STEM and ARPES measurements showed that there are many parallel mirror twin boundaries (MTBs) composed of Re_4_ clusters in the electrochemically exfoliated monolayer ReS_2_ plane, which separate two crystal domains with translational dislocations in the [010] direction (Figure 7e). Such fully parallel MTBs were not found in the monolayer ReS_2_ prepared by CVD [74], and the density of MTBs increased with the size of the intercalated molecules. More importantly, the monolayer ReS_2_ obtained by the intercalation of large-size molecules (THA) showed a ferromagnetic transition at 60 K, which was not present in the ReS_2_ crystals and the ReS_2_ monolayers obtained by the intercalation of small-size molecules (TPA). They believe that the stable strain and S vacancies in MTBs induced by large-size molecular intercalation contribute to the generation of ferromagnetism in monolayer ReS_2_ (Figure 7f).

Magnetic anisotropy in 2D magnetic materials can also be modulated by interlayer chemical modulation. It is well known that magnetic anisotropy plays a key role in the stable existence of the magnetic properties of 2D materials. Most of the 2D magnetic materials found so far, such as 1T-CrTe_2_ and Fe_4_GeTe_2_, are characterised by in-plane magnetic anisotropy [75,76]. A completely different perpendicular magnetic anisotropy (PMA) from the pristine CrTe_2_ was recently observed in Cr-intercalated CrTe_2_ [77]. Cr-intercalated CrTe_2_ crystals were synthesised by the indirect oxidation of K_x_Cr_1.3_Te_2_ and iodine in acetonitrile, and Cr-CrTe_2_ flakes were obtained by mechanical exfoliation. The XRD results show that the intercalated Cr atoms were distributed in the interstices between the CrTe_2_ layers. By magnetic measurements, they found that the Curie temperature of the Cr-CrTe_2_ flakes is greater than 306 K. Furthermore, when the external magnetic field is perpendicular to the ab plane of Cr-CrTe_2_, the magnetisation more easily reaches saturation, which indicates that Cr-CrTe_2_ has PMA. In addition, Hall transport measurements also show that Cr-CrTe_2_ has the largest anomalous Hall factor at present. These excellent properties provide great potential for the application of Cr-CrTe_2_ in the field of spintronic devices.

Apart from introducing ferromagnetism in non-magnetic materials, intrinsic two-dimensional magnetic materials can also be regulated by the intercalation method to optimise their magnetic properties. Fe_3-x_GeTe_2_ is a metallic layered magnetic material with high Curie temperature and strong magnetic anisotropy, which is a favourable candidate for room-temperature-stable two-dimensional magnetic materials [78]. Recently, Deng et al. successfully obtained ferromagnetic single-layer Fe_3_GeTe_2_ and increased the Curie temperature of Fe_3_GeTe_2_ atomic flakes to room temperature by gated lithium ion intercalation [79]. Due to the weak intralayer bonding, Fe_3_GeTe_2_ flakes are easily broken in the traditional mechanical exfoliation process, resulting in small-size Fe_3_GeTe_2_ flakes. Therefore, they developed an Al_2_O_3_-assisted exfoliation method to successfully exfoliate large-size Fe_3_GeTe_2_ monolayers due to the higher affinity and larger contact area between Al_2_O_3_ and Fe_3_GeTe_2_. The Curie temperature of Fe_3_GeTe_2_ increases with the increase in thickness, and the Curie temperature of single-layer Fe_3_GeTe_2_ is about 20 K. They used exfoliated Fe_3_GeTe_2_ atomic flakes to construct iFET devices for lithium ion intercalation. The ferromagnetic transition temperature regulated by V_g_ is determined by measuring the anomalous Hall effect. The Curie temperature of the Fe_3_GeTe_2_ flakes can be significantly adjusted by changing the electron doping and reaching a maximum of about 300 K at V_g_ = 1.75. They believe that the extreme electron doping (~1.5 × 10^14^ cm^−2^ per layer) caused by gated intercalation leads to a significant shift in the electronic band of Fe_3_GeTe_2_. The doped electrons gradually fill the subbands formed by Fe d_z_^2^, d_xz_, and d_yz_ orbitals, increasing the density of the states of the Fermi level, which may be the reason for the sharp increase in Curie temperature.

**Figure 8:**
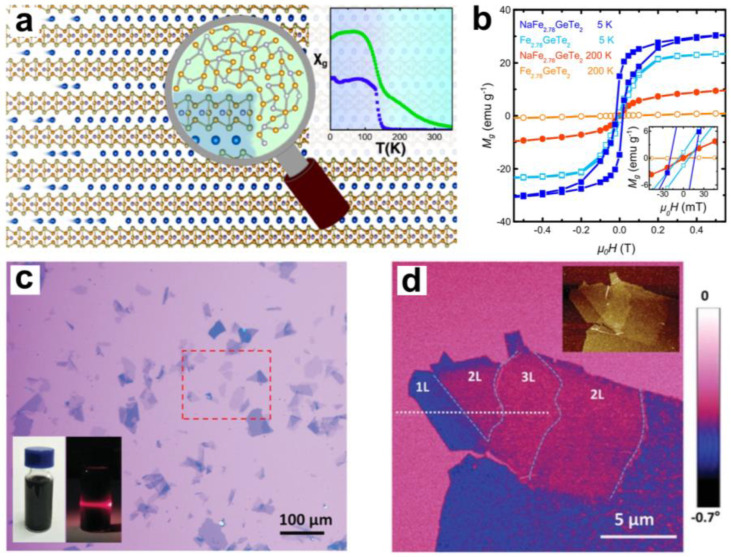
(**a**) Schematic diagram of Na intercalation layer Fe_2.78_GeTe_2_ generating amorphous impurities. Reproduced with permission from Ref. [80]. Copyright 2019, American Chemical Society. (**b**) M−H curves of Fe_2.78_GeTe_2_ and NaFe_2.78_GeTe_2_ at different temperatures. Reproduced with permission from Ref. [80]. Copyright 2019, American Chemical Society. (**c**) Large-size VSe_2_ flakes under optical microscope. Reproduced with permission from Ref. [81]. Copyright 2019, Wiley−VCH. (**d**) Magnetic force microscopy images of VSe_2_ flakes of different thicknesses. Reproduced with permission from Ref. [81]. Copyright 2019, Wiley−VCH.

Later, Weber et al. also observed room-temperature ferromagnetism in Na-intercalated Fe_2.78_GeTe_2_ powder, which is entirely different from the above situation [80]. The impurities generated by the intercalation wholly cause the room temperature ferromagnetism in NaFe_2.78_GeTe_2_, and there is no increase in Curie temperature. NaFe_2.78_GeTe_2_ was prepared by Na/benzophenone (Ph_2_CO) and Fe_2.78_GeTe_2_ through Na reductive intercalation in tetrahydrofuran (THF). The PXRD, transmission electron microscope (TEM), and energy-dispersive X-ray spectroscopy (EDX) measurements show that there are Na_2_Te and ferromagnetic amorphous Fe_2-x_Ge impurities in the prepared samples, and an increase in the impurity phase ratio after annealing also indicates that the decomposition of NaFe_2.78_GeTe_2_ causes the amorphous impurity (Figure 8a). Through magnetic measurements, they found that Fe_2.78_GeTe_2_ exhibits paramagnetism at 200 K, whilst NaFe_2.78_GeTe_2_ containing Na_2_Te and Fe_2-x_Ge still has a hysteresis loop at 200 K (Figure 8b), even at 350 K. However, the sample’s magnetisation at 300 K is very close to the expected magnetisation of the Fe_2-x_Ge impurity in the sample. Moreover, the specific susceptibility of the samples before and after intercalation shows a transition near 150 K, which indicates that the Curie temperature is almost unchanged. Therefore, they believe that the impurity Fe_2-x_Ge causes room-temperature ferromagnetism in NaFe_2.78_GeTe_2_. More interestingly, the higher electron doping level (~6.95 × 10^14^ cm^−2^ per layer) caused by Na intercalation compared with gated intercalation does not increase the Curie temperature of powder NaFe_2.78_GeTe_2_. The different properties of the intercalation method in materials of different dimensions indicate its potential to explore complex singular effects and new coupling mechanisms. It also shows the effectiveness of the intercalation method in introducing carrier doping.

Intercalation can also be used as a more effective exfoliation and modulation method in some difficult-to-handle layered magnetic materials. 1T-VSe_2_ is a layered magnetic material. Its narrow interlayer spacing and the easy oxidation of the single layer make it difficult to obtain a ferromagnetic single-layer VSe_2_ by mechanical exfoliation. Furthermore, then, there is some controversy about the existence of intrinsic ferromagnetism in monolayer VSe_2_ [82,83,84]. Recently, Yu et al. observed strong ferromagnetism at room temperature in monolayer 1T-VSe_2_ obtained by electrochemical exfoliation [81]. The monolayer VSe_2_ was obtained by organic cation intercalation exfoliation and thiol-passivation. Figure 8c shows a ultra-thin VSe_2_ flake with a size of more than 100 microns under an optical microscope. The ultra-thin VSe_2_ flake after thiol passivation has good environmental stability, and the magnetic signal of the VSe_2_ flake can be observed at room temperature using magnetic force microscopy (Figure 8d). Magnetic measurements show that monolayer VSe_2_ exhibits significant hysteresis lines at both 10 K and 300 K, and the Curie temperature of monolayer VSe_2_ is as high as 470 K, exhibiting excellent 2D ferromagnetic properties. Interestingly, the ferromagnetism of VSe_2_ also shows a concentration dependence of Se vacancies, and their experiments suggest that Se vacancies can enhance the ferromagnetism of VSe_2_. However, the thiols used to protect the surface molecules have occupied the vacancies of Se in VSe_2_, so they concluded that the monolayer VSe_2_ is intrinsically ferromagnetic and that the ferromagnetism of the experimentally passivated monolayer VSe_2_ is the lower limit of the ferromagnetism of the material. Therefore, introducing microscopic interactions through interlayer chemical regulation is a promising way to obtain new 2D magnetic materials.

## 4. Interlayer Chemical Modulation of Lattice Phase Transitions

**Figure 9:**
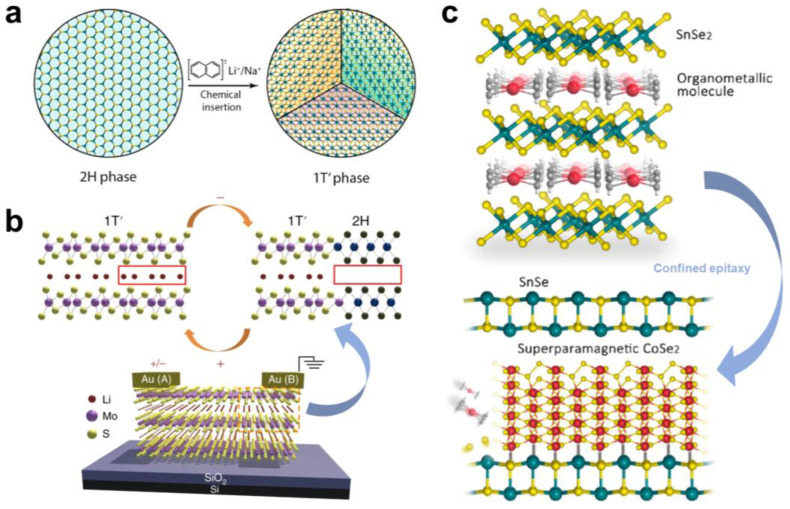
(**a**) Schematic diagram of the 1T’-MoS_2_ lattice structure with three orientations formed after lithium intercalation. Reproduced with permission from Ref. [86]. Copyright 2016, American Chemical Society. (**b**) Schematic diagram of the 2H-1T’ phase transition in the MoS_2_ lattice by controlling the flow of Li^+^ ions. Reproduced with permission from Ref. [90]. Copyright 2018, Springer Nature. (**c**) Schematic diagram of vdW epitaxial growth of CoSe_2_-SnSe heterostructure. Reproduced with permission from Ref. [92]. Copyright 2022, American Chemical Society.

The lattice structure of solid materials determines the physical properties. The insertion of atoms or molecules through interlayer chemical modulation can introduce strain or chemical bonds into the material, which can induce lattice distortion and change the properties of the host material. More interestingly, by controlling the intercalation kinetic factors of the guest species, the local intercalation and cyclic intercalation of the material can even be carried out to realise the cyclic phase transition of the lattice structure, further enriching the application prospect of layered materials. For example, Li intercalation will inject electrons into MoS_2_, resulting in a 2H→1T′ structural transition, and the de-intercalation of Li ions will also lead to some of the 1T′ phase to reconvert into the 2H phase. This cyclic phase transition is an ideal choice for lithium-ion battery electrode materials. However, when MoS_2_ is used as an electrode material, it will quickly break up in the structural disorder and uneven phase transition due to the repeated insertion and de-intercalation of Li ions and cannot maintain good cycle stability [85]. Leng et al. pre-intercalated MoS_2_ by liquid phase intercalation and obtained 1T’-Li_1.0_MoS_2_ with good cycle stability (Figure 9a) [86]. Compared with the MoS_2_ electrode, the Li_1.0_MoS_2_ electrode has a capacity of 636 mA h g^−1^ at 1 A g^−1^ and still has 80% of the initial capacity after 2000 cycles. High-resolution STEM annular dark-field (ADF) imaging shows that the 2H→1T’ phase transition induced by Li intercalation results in the formation of interlaced, interconnected 1T’-MoS_2_ nanodomains with different orientations in Li_1.0_MoS_2_. These nanodomains can undergo stable recombination in the phase transition process Li_x_MoS_2_ + (4 − x)Li^+^ + (4 − x)e^−^ ⇔ Mo + 2Li_2_S during multiple charge–discharge cycles. This is the reason for its good cycle stability, and the domain boundaries of these crystal domains also provide more channels for ion transport during charge and discharge, further improving the battery capacity.

The lattice transition of MoS_2_ also shows effective modulation on thermoelectric properties. Ng et al. studied the thermoelectric properties of the mixed-phase material Li_x_MoS_2_ dominated by the 1T’ phase by vacuum thermal annealing [87]. They found that, as the number of annealing cycles increases, the Raman spectrum results show a structural phase transition of 1T’→2H, which is due to Li ion de-intercalation under thermal annealing. At the same time, the Seebeck coefficient of Li_x_MoS_2_ continuously decreases from 20 μV K^−1^ to reverse saturation −290 μV K^−1^, which means that the major charge carrier changes from p-type to n-type. The initial p-type conduction characteristic of Li_x_MoS_2_ is caused by 1T′ Li_x_MoS_2_, similar to the previous thermoelectric research results of 1T MoS_2_ [88]. Therefore, they attribute the p-type conduction to the Fermi level in the mixed phase Li_x_MoS_2_ deeper into the valence band of the semimetal 1T′ Li_x_MoS_2_. The n-type conductivity and the decreasing Seebeck coefficient is associated with the defects, strain, and inhomogeneous 2H phase distribution due to the thermal annealing. In addition, the XPS results show that the sample obtained after the 26th annealing only contains a small amount of 1T’ phase, but its thermoelectric power factor is only about 27 μW m^−1^ K^2^. In contrast, the pure 2H-MoS_2_ can exhibit a much larger thermoelectric power factor. These results mean that the non-uniform phase transition caused by the de-intercalation leads to the disorder of the lattice structure [89]. Therefore, it is still difficult to achieve a fully reversible phase transition of 2H⇔1T′ by ion insertion and de-intercalation, and it is crucial to eliminate or reduce the disordered structure introduced by the intercalation and de-intercalation processes. However, recent studies have shown that the reversible local phase transition induced by intercalation does not introduce disordered structures, which provides an alternative idea to realise devices with specific functions. Zhu et al. realised MoS_2_ thin films with locally reversible phase transition by electric field control and constructed neurosynaptic devices [90]. They constructed Au/MoS_2_/Au devices using 2H-MoS_2_ thin films, and embedded Li^+^ ions into 2H-MoS_2_ in the device through the liquid-phase intercalation method to achieve 2H-1T′ phase transition (Figure 9b). By applying an alternating in-plane bias, the device shows a memristor property of alternating transition from a high-resistance state (HRS) to a low-resistance state (LRS), which is due to the alternating phase transition of 2H-1T′ in the local region caused by the flow of interlayer Li^+^ ions. Moreover, applying positive and negative alternating short pulses to the device can simulate the excitation and inhibition of synapses, and this alternating phase transition shows good durability. In addition, the layered structure of MoS_2_ also provides a low-activation-energy channel for Li^+^ ions, which makes Li^+^ have a high diffusion rate in MoS_2_ and facilitates the fabrication of high-performance synaptic devices [91]. Therefore, based on the controllable in-plane Li^+^ ions exchange in Li_x_MoS_2_, they constructed a low-power neural network synaptic device with a shared electrode. By programming the bias voltage of four Au/Li_x_MoS_2_/Au devices, the flow of Li^+^ is regulated, and the synaptic cooperation and competition phenomena of biological nerves are simulated. It is also expected that this strategy can be applied to other layered 2D-MCs with multiphase structures, opening a new way to exploit neuromorphic devices.

In addition, the heterophase filling (HPF) system with heterogeneous interfaces and boundaries formed by introducing chemical bonds between layers through intercalation has recently proved to be a promising way to develop new thermoelectric materials. Zhao et al. developed a vdW confined epitaxial method to prepare a SnSe-CoSe_2_ superlattice structure with ultra-low in-plane thermal conductivity [92]. The SnSe_2_-Co(C_5_H_5_)_2_ superlattice was prepared by liquid-phase intercalation, and then SnSe-CoSe_2_ heterostructure was formed by the reaction of SnSe_2_ with Co(C_5_H_5_)_2_ in the layer under the heating condition of an inert atmosphere (Figure 9c). Through electrical and thermal transport measurements, they found that SnSe-CoSe_2_ maintains the p-type characteristics of SnSe crystals and has higher conductivity than polycrystalline SnSe at 850 K, which indicates that the SnSe layer of the superlattice has good crystalline integrity and retains good in-plane conductivity. At the same time, the in-plane thermal conductivity of SnSe-CoSe_2_ at room temperature and 850 K is 0.65 W m^−1^ K^−1^ and 0.27 W m^−1^ K^−1^, respectively, which is the lowest in-plane thermal conductivity of SnSe-based materials, even lower than many traditional low-thermal-conductivity thermoelectric materials [93]. In addition, SnSe is nonmagnetic, whilst the SnSe-CoSe_2_ “S”-shape M−H curve and negligible coercivity at 380 K indicate that CoSe_2_ is superparamagnetic. This superparamagnetism has been shown to enhance phonon scattering [94]. Therefore, they believe that, based on the scattering of phonons by abundant grain boundary and lattice distortion in SnSe-CoSe_2_, the superparamagnetic fluctuation and random rotation of magnetic domains of superparamagnetic nanocrystals CoSe_2_ further increase the scattering of phonons, which together lead to ultra-low in-plane thermal conductivity in SnSe-CoSe_2_. This confined epitaxial growth based on the intercalation method provides another idea for preparing 2D heterojunctions.

## 5. Conclusions and Outlook

In summary, we review the research progress of interlayer chemical modulation on 2D-MCs involving superconducting transition, CDW transition, semiconductor–metal transition, magnetic transition, and lattice transition in recent years. Due to the unique interlayer guest–host interactions, the interlayer chemical modulation presents an ideal approach to control the phase transitions in 2D layered MCs, effectively regulating their electronic structure, spin coupling, and lattice distortion. As a result, numerous exciting phenomena and a new understanding of the electronic correlation in many-body states were demonstrated. In addition, the interlayer chemical modulation of phase transitions in 2D-MCs shows great application potential in the fields of spintronic devices, information processing, and energy conversion. Although current research toward the interlayer chemical modulation of 2D materials has made great progress, there are still some problems worthy of further research. For example, currently, most of the guest species modulated by interlayer chemistry are still concentrated in alkali or alkaline earth metals, and there are few studies on other types of guest species (such as transition metal atoms, organic molecules, high-valent cations, or anions). The guest species with complex electronic structures may provide more abundant interlayer coupling, resulting in new phenomena and principles. Meanwhile, artificial 2D vdW heterojunction materials with peculiar interlayer interactions also provide a new direction for selecting intercalation host materials, which may result in more exciting physical properties. In addition, it is also essential to further explore the potential applications of new phenomena caused by intercalation.

## Data Availability

Not applicable.

## Sample Availability

Samples of the compounds are not available from the authors.
